# A systematic review and meta-analysis of the effects of early mobilization therapy in patients after cardiac surgery: A protocol for systematic review

**DOI:** 10.1097/MD.0000000000018843

**Published:** 2020-01-24

**Authors:** Bin Chen, Xiaofang You, Yuan Lin, Danyu Dong, Xuemin Xie, Xinyi Zheng, Dong Li, Wanqing Lin

**Affiliations:** Department of Rehabilitation, The Affiliated People's Hospital of Fujian University of Traditional Chinese Medicine, Fuzhou 350004, Fujian Province, China.

**Keywords:** mobilization, cardiac surgery, intensive care unit, protocol, review

## Abstract

**Background:**

Prolonged hospitalization and immobility of critical care patients elevates the risk of long-term physical and cognitive impairments. However, the therapeutic effects of early mobilization have been difficult to interpret due to variations in study populations, interventions, and outcome measures. This systematic review and meta-analysis aims to assess the effects of early mobilization therapy for non-emergency cardiac surgery patients in the intensive care unit (ICU).

**Methods:**

The following databases will be used to search for relevant keywords: PubMed, Embase, CINAHL, PEDro, and the Cochrane Library from inception to September 2018 by 2 researchers independently. Randomized controlled trials (RCTs), will be included if patients are adults (≥18 years) admitted to any ICU for cardiac surgery due to cardiovascular disease and who are treated with experimental physiotherapy initiated in the ICU (pre, post, or perioperative). The Review Manager 5.3 will be used for meta-analysis and the evidence level will be assessed by using the method for Grading of Recommendations Assessment, Development, and Evaluation (GRADE). Continuous outcomes will be presented as the weighted mean difference (WMD) or standardized mean difference (SMD) with 95% confidence interval (CI), while dichotomous data will be expressed as relative risk (RR) with 95% CI. If the included studies have existing heterogeneity (P < 0.1), a random-effects model will be used. Otherwise, we will calculate using a fixed effects model.

**Results:**

This review will evaluate the effects of early mobilization on length of ICU and hospital stay, physical function and adverse events in patients with cardiac surgery patients in the ICU.

**Conclusion:**

This systematic review will comprehensively provide conclusive evidence of the therapeutic effect of early mobilization on cardiac surgery patients in the ICU.

PROSPERO Research registration identifying number: CRD42019135338.

## 1 Introduction

Prolonged intensive care hospitalization has been linked to increased morbidity and long-term mortality after hospital discharge.^[1]^ It has been estimated that up to 46% of intensive care unit (ICU) patients acquire ICU-acquired weakness (ICU-AW) during their stay.^[2]^ ICU-AW includes polyneuropathy, myopathy, and/or muscular atrophy which can prolong immobilization and inhibit long-term physical and cognitive function.^[3]^ Early physical rehabilitation has been associated with improved physical function and is recommended for ICU patients by the European Society of Intensive Care Medicine.^[4]^ While independent studies have reported a variety of benefits of early mobilization therapy, including reduced mechanical ventilation days, reduced hospital length of stay, and functional outcomes,^[5–7]^ various reviews have confirmed only the short-term benefits of early mobilization intervention, calling into question whether the high resource and labor costs offset these short-term benefits.^[8–10]^

Other reviews of early mobilization therapy in critically ill patients have yielded conflicting findings, with either no or inconsistent effects on functional recovery, quality of life, length of ICU or total hospitalization stay, and long or short-term mortality.^[11,12]^ Conflicting findings may be due to several factors including intervention differences, variations in reporting, quality of available resources, etc.^[13]^ In fact, systematic or quantitative reviews of the literature have thus far mostly concluded that there is insufficient evidence of the effect of early physiotherapy on the physical improvement of critically ill adults.^[14]^ Moreover, it should be noted that some systematic reviews have entirely deemed the current body of literature suboptimal for comparison due to lack of consistency or reliability in the delivered intervention.^[15,16]^ For example, Reid et al^[15]^ report that out of 117 studies evaluated, none reported the same intervention in exactly the same way. Thirty-seven percent did not report intervention start time and 26% did not report overall intervention duration, limiting understanding and generalizability of the interventions. Another potentially confounding factor is the variety of patient populations (and acuities) evaluated across studies of ICU early mobilization, which often include patients admitted for cardiac disease, respiratory illness, and acquired brain injury, among other critical illnesses. Toward the aim of improving homogeneity of patient populations, an increasing number of targeted studies are being undertaken.

It has been reported that 58% of cardiac surgery patients are vulnerable to postoperative complications and subsequent delays in hospital discharge and functional recovery. While currently, early mobilization and prophylactic respiratory physiotherapy are postoperatively prescribed for cardiac surgery patients, no consensus exists regarding optimal mobility protocols nor how these interventions impact hospitalization duration, postoperative complications, or functional recovery of cardiac surgery patients specifically. To date, Ramos Dos Santos et al is one of the only evaluations of the existing literature on the effect of early mobilization in patients after nonemergency cardiac surgery. Their systematic review of open-heart surgery patients across a variety of “early mobilization” interventions found that early mobilization improved functional outcomes for patients compared with untreated (bed rest) patients. In 2 of 5 studies including a control group, hospital length of stay was significantly reduced, however, lack of homogeneity between early mobility protocols precluded a meta-analysis, hindering an effective conclusion regarding the evidence of therapy on cardiac surgery patients in the ICU.^[17]^

To address the lack of conclusive evidence of the effect of early mobilization on cardiac surgery patients in critical care settings, this systematic review and meta-analysis aimed to evaluate randomized controlled trials (RCTs) exclusively evaluated in cardiac surgery patients treated experimentally with early mobilization. Due to variations in mobilization protocols and ancillary interventions in the body of literature, the purpose of this study was a focused, quantitative assessment of the effect of early mobilization on some of the most widely reported risk factors of ICU-AW and associated complications, length of ICU, and hospital stay. This report sheds light on one of the prevailing questions regarding the effect of early mobilization intervention in critically ill patients by assessing a targeted population filtered by stringent inclusion criteria.

## 2 Methods

### 2.1 Study registration

The protocol of this study has been registered in PROSPERO (CRD42019135338) at https://www.crd.york.ac.uk/PROSPERO/. The Preferred Reporting Items for Systematic Reviews and Meta-analyses Protocols (PRISMA-P) statement was the guideline during the design of this study.

### 2.2 Inclusion criteria for study selection

#### 2.2.1 Type of study

We will estimate the research literature according to the criteria of the review objectives and participants, interventions, comparisons, outcomes. RCTs will be included if patients are adults (≥18 years) admitted to any ICU for cardiac surgery due to cardiovascular disease and who are treated with experimental physiotherapy initiated in the ICU (pre-, post-, or perioperative). We will include such studies if the expression “randomization” is mentioned. However, we will grade these studies as high in the “risk of bias assessment” if the detailed description on the randomization process is not provided. Furthermore, if an incorrect randomization method such as coin toss was used, the study will not be included. Additionally, the language of the publications will be limited to English.

#### 2.2.2 Types of participants

The adult patients (≥18 years) admitted to any ICU for cardiac surgery due to cardiovascular disease and who are treated with experimental physiotherapy initiated in the ICU (pre-, post-, or perioperative).

#### 2.2.3 Types of interventions and comparators

Interventions will include passive or active exercises, strengthening exercises, cycling, progressive mobility, or any combination thereof. Studies will be included only if a comparator group includes either no prescribed mobilization intervention or delayed intervention (i.e., intervention prescribed after ICU discharge).

#### 2.2.4 Types of outcome measures

Length of ICU and hospital stay will be evaluated as the primary outcomes. Physical function and adverse events will be evaluated as the secondary outcomes.

1. Physical function: The ability to perform everyday activities such as basic ADLs) as measured by a validated scale (e.g., Barthel index, functional independence measure) or physical performance tasks (as measured by a scale such as the Physical Function ICU Test, Acute Care Index of Functional Status, Short Physical Performance Battery, walking tests.
2. Adverse events: Falls, accidental dislodgement of attachments, hemodynamic instability, oxygen desaturation, or any other adverse events defined by study authors.

### 2.3 Data sources

The following databases will be used to search for relevant keywords: PubMed, Excerpta Medica database (Embase), Cumulative Index of Nursing and Allied Health Literature (CINAHL), Physiotherapy Evidence Database (PEDro), and the Cochrane Central Register of Controlled Trials. The search will be performed for studies from inception to September 20, 2018.

### 2.4 Search strategy

The strategy will be created according to the Cochrane handbook guidelines. The established search strategy for PubMed was displayed, as follows:

Mesh term #1: ((mobilisation) OR (mobilization) OR (ambulation) OR (exercise) OR (physical therapy) OR (physiotherapy) OR (fitness training) OR (strength training) OR (stretching) OR (manipulation) OR (rehabilitation)): ti, ab, kw

Mesh term #2: ((intensive care) OR (critical care) OR (intensive care unit) OR (ICU) OR (critical illness)): ti, ab, kw

Mesh term #3: cardiac surgery: ti, ab, kw

Mesh term #4: ((clinical trials) OR (randomized controlled trials))

#1 AND #2 AND #3 AND #4

### 2.5 Data collection and analysis

#### 2.5.1 Selection of studies

**Figure 1:**
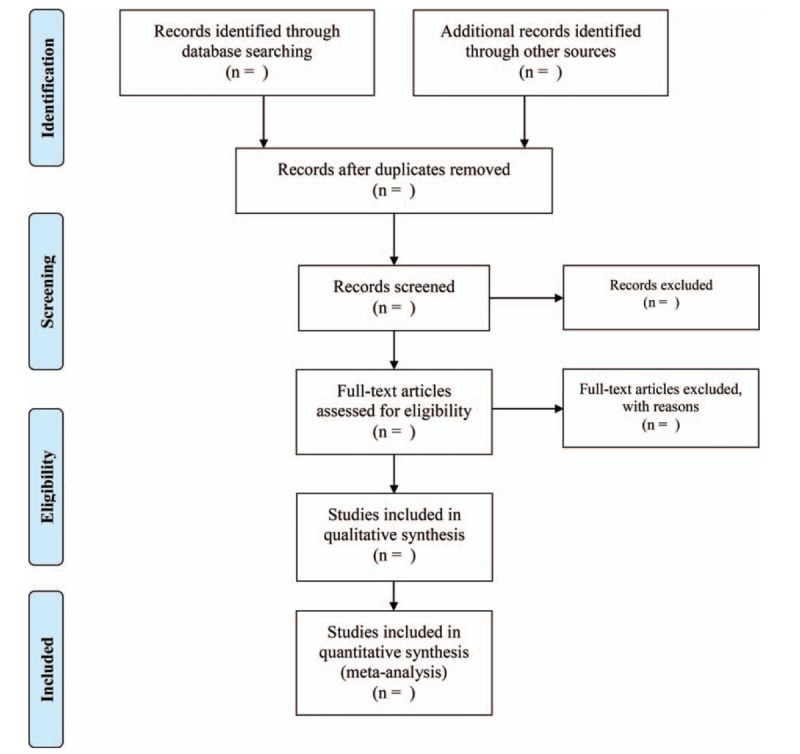
PRISMA flow diagram of study process.

The researchers will import the retrieved literature into an EndNote library and eliminate duplicate data. Two review authors (XFY and DYD) will select studies for eligibility and check against the inclusion criteria independently. Any disagreement will be resolved by consensus or consultation with a third independent researcher (YL). The selection process is illustrated in a PRISMA flow diagram (Fig. 1).

#### 2.5.2 Data extraction and management

Data will be extracted by 2 reviewers (XYZ and DL) independently using a preconstructed data extraction form. The data extraction form includes: the publication information (title, authors, year, etc), participant characteristics (age, gender, etc), intervention details (intervention of experimental group and intervention of control group, frequency, intensity, duration, follow-up), outcomes (primary outcome and secondary outcome, outcome instruments), study design (randomized, blinded, etc), adverse events, and other detailed information. If necessary, we will contact the corresponding authors of trials as much as possible for further information.

#### 2.5.3 Assessment of risk of bias and reporting of study quality

The Cochrane risk of bias tool will be applied to evaluate the quality and risk of bias in the ultimately included studies by 2 authors (XFY and DYD) independently.^[18]^ Risk of bias assessment categories will include the following: random sequence generation; allocation concealment; blinding of participants; blinding of outcome assessors; completeness of outcome data; selective outcome reporting; and other biases. The assessments for each item will be graded as low risk, unclear risk, and high risk to evaluate several risks of bias that can occur in RCTs. In the case of other sources of bias, it was evaluated as “low” if the characteristics of participants in each group were reported to be statistically homogeneous at baseline, but was otherwise rated “high.” The results were presented as a risk of bias graph and risk of bias summary using the Cochrane Collaboration's software program Review Manager (RevMan) version 5.3 for Windows (Copenhagen, The Nordic Cochrane Centre, the Cochrane Collaboration, 2012). If there is any disagreement take place, the arbiter (YL) will do the final judge.

#### 2.5.4 Measures of treatment effect

For all dichotomous variables, relative risks or odds ratios with 95% confidence intervals (CIs) will be presented. For all continuous variables, mean difference (MD) or standard MDs (SMDs) with 95% CI will be calculated. When the same outcome is measured in different ways, the SMD with 95% CI will be selected to express the size of the intervention effect. Two-sided *P-*value of <.05 is defined as statistical significance.

#### 2.5.5 Dealing with missing data

If there are missing data, we will attempt to contact the corresponding authors of included studies for any necessary data by e-mail. However, if the missing data cannot be obtained, the study will be excluded from the analysis.

#### 2.5.6 Assessment of heterogeneity

The *I*^2^ statistic and Chi-squared test will be used to assess statistical heterogeneity, with *P* < .1 of Chi-squared test or *I*^2^ > 75% suggesting high statistical heterogeneity among the studies. If the included studies have existing heterogeneity, a random-effects model will be used. Otherwise, we will use a fixed-effects model for calculation.

#### 2.5.7 Assessment of reporting bias

If more than 10 studies are included, visual asymmetry on the funnel plots will be used to assess the potential reporting biases. In addition, we will test asymmetry using the Harbord modified test for dichotomous outcomes and Egger test for continuous outcomes.

#### 2.5.8 Data synthesis

The Review Manager 5.3 will be employed for meta-analysis. When statistical heterogeneity is low among the results, the fixed-effects model will be used for the meta-analysis. However, there is considerable heterogeneity, the random-effects model will be performed to analyze the pooled effect estimates.

#### 2.5.9 Subgroup analysis

If there are significant clinically and statistically heterogeneous (*I*^2^ > 75%) and enough RCT studies, subgroup analysis will be carried out. According to the types of interventions and different outcomes, the origins of heterogeneity will be carried out.

#### 2.5.10 Sensitivity analysis

A sensitivity analysis will be conducted to identify whether the review conclusions are robust according to the following criteria: missing data, sample size, heterogeneity qualities, and statistical model.

#### 2.5.11 Grading the quality of evidence

The evidence level will be assessed by using the method for Grading of Recommendations Assessment, Development, and Evaluation (GRADE) and classified into 4 possible ratings: very low, low, moderate, or high.

## 3 Discussion

Length of hospital stay has been widely correlated with risk of postoperative complications, in-hospital mortality, and medical costs of patients undergoing cardiac surgery.^[19]^ Increased ICU stay has also been linked to increased risk of myopathy, neuropathy with can negatively impact cognition and mobilization in the long term.^[3]^ Many hospitals are investing in early mobilization/physiotherapy interventions to reduce these adverse events. To date, early mobilization protocols remain nonstandardized and are thereby difficult to evaluate. This is exacerbated by the variability of the patient acuities that make up the collective population in the majority of robust evaluations of efficacy. Though widely reported to be safe and beneficial, much literature has challenged the universal endowment of early mobility to reduce hospitalization or enhance patient outcomes long term. This systematic review and meta-analysis used a stringent inclusion criteria and narrow population of hemodynamically stable patients undergoing cardiac surgery to reduce some of the inconsistencies that plague the current body of literature. It will provide high-quality evidence-based medicine to determine whether the early mobilization therapy is an effective and safe intervention for nonemergency cardiac surgery patients in the ICU.

The ethical approval will not be needed because no primary data are collected. Our results will provide clear evidence to determine whether the early mobilization therapy is an effective and safe intervention for nonemergency cardiac surgery patients in the ICU, and thus will be beneficial to patients, practitioners, and policy makers. This review will be published in a peer-reviewed journal and will be presented at an international academic conference for dissemination.

## Author contributions

**Conceptualization:** Bin Chen, Wanqing Lin.

**Methodology:** Xiaofang You, Yuan Lin, Danyu Dong, Xuemin Xie, Xinyi Zheng, Dong Li.

**Supervision:** Bin Chen, Yuan Lin.

**Writing – original draft:** Bin Chen, Xiaofang You.

**Writing – review & editing:** Bin Chen, Xiaofang You, Wanqing Lin.

Bin Chen orcid: 0000-0002-2459-1605.
